# Oxidative Stress in Hemodialysis Patients: A Review of the Literature

**DOI:** 10.1155/2017/3081856

**Published:** 2017-09-12

**Authors:** Vassilios Liakopoulos, Stefanos Roumeliotis, Xenia Gorny, Evangelia Dounousi, Peter R. Mertens

**Affiliations:** ^1^Division of Nephrology and Hypertension, 1st Department of Internal Medicine, AHEPA Hospital, School of Medicine, Aristotle University of Thessaloniki, Thessaloniki, Greece; ^2^Clinic of Nephrology and Hypertension, Diabetes and Endocrinology, Otto von Guericke University Magdeburg, Magdeburg, Germany; ^3^Department of Nephrology, School of Medicine, University of Ioannina, Ioannina, Greece

## Abstract

Hemodialysis (HD) patients are at high risk for all-cause mortality and cardiovascular events. In addition to traditional risk factors, excessive oxidative stress (OS) and chronic inflammation emerge as novel and major contributors to accelerated atherosclerosis and elevated mortality. OS is defined as the imbalance between antioxidant defense mechanisms and oxidant products, the latter overwhelming the former. OS appears in early stages of chronic kidney disease (CKD), advances along with worsening of renal failure, and is further exacerbated by the HD process per se. HD patients manifest excessive OS status due to retention of a plethora of toxins, subsidized under uremia, nutrition lacking antioxidants and turn-over of antioxidants, loss of antioxidants during renal replacement therapy, and leukocyte activation that leads to accumulation of oxidative products. Duration of dialysis therapy, iron infusion, anemia, presence of central venous catheter, and bioincompatible dialyzers are several factors triggering the development of OS. Antioxidant supplementation may take an overall protective role, even at early stages of CKD, to halt the deterioration of kidney function and antagonize systemic inflammation. Unfortunately, clinical studies have not yielded unequivocal positive outcomes when antioxidants have been administered to hemodialysis patients, likely due to their heterogeneous clinical conditions and underlying risk profile.

## 1. Introduction

Oxidative stress (OS) is defined as a disruption of the balance between oxidative products and antioxidant defense mechanisms, with the former overwhelming the latter [1]. Oxidative molecules include reactive oxygen species (ROS) and nitrogen species, such as nitric oxide (NO), while antioxidants can be endogenous or dietary/exogenously administrated molecules. OS triggers the oxidation and subsequent damage of molecules, such as proteins, lipids, nucleic acids (DNA), and carbohydrates. OS is implicated in the pathologic pathways of various conditions, such as diabetes mellitus (DM), atherosclerosis, inflammation, and progression of chronic kidney disease (CKD) to end-stage renal disease (ESRD). OS is very common in CKD; it is present even in the early stages, gradually increases along with renal impairment, and is further exacerbated by hemodialysis (HD) procedures. CKD is accompanied by pronounced oxidation of proteins, carbohydrates, and lipids, leading to lipid peroxidation and the accumulation of advanced glycation end products (AGEs) which cause severe tissue damage. Also, OS has been associated with renal anemia, malnutrition, *β*2-microglobulin amyloidosis, and cardiovascular disease (CVD) and is an independent predictor of mortality and morbidity in this group of patients [1, 2].

## 2. CKD and OS

There is a growing body of evidence showing that CKD patients are characterized by enhanced OS, even in early stages [3, 4]. OS increases in later stages of CKD and becomes more severe in end-stage renal disease patients undergoing maintenance patients [5]. It has been shown that the presence of OS may cause dramatic modifications in the normal kidney, similar to those seen in CKD. Oxidant molecules contribute to progressive kidney damage by promoting renal ischemia, by inciting glomerular injury, cell death, and apoptosis, and finally by stimulating a severe inflammatory process [6, 7]. Moreover, OS is a major contributor to several conditions which predispose to CKD, such as diabetes, hypertension, and atherosclerosis promoting indirectly the progression of renal damage [6]. In kidney failure, accumulation of ROS or reduction in antioxidant systems can be observed [6]. Specifically, accumulation of ROS, especially O_2_^−^, leads to NO inactivation and deficiency, which is a critical antioxidant that protects kidney function by increasing renal blood flow, enhancing pressure natriuresis, regulating tubuloglomerular function, and preserving fluid and electrolyte homeostasis. NO deficiency and high levels of plasma O_2_^−^ are considered critical promoters of OS. Several *in vivo* studies highlighted that CKD is a state of NO deficiency: hypertensive animal models showed increased levels of O_2_^−^ in the endothelium and kidneys; animals with NO deficiency developed salt retention, hypertension, albuminuria, and glomerulosclerosis; and oral intake of the NO precursor molecule L-arginine in nephrectomized rats increased estimated glomerular filtration rates (eGFR) and improved glomerular function [6, 8, 9]. Chen et al. showed that plasma levels of O_2_^−^ were significantly increased in maintenance hemodialysis patients compared to healthy controls [10].

Many studies concluded that NO deficiency due to inactivation by O_2_^−^ or reduced renal NO production contributes to CKD progression [6]. Yilmaz et al. showed that red blood cells (RBCs) from patients in early stages of CKD (1-2) present enhanced OS status compared to healthy subjects [11]. Furthermore, OS markers were strongly inversely associated with eGFR and were progressively augmented, along with deterioration of renal function [11]. Similar results were reported by Terawaki et al. In a cohort of 55 nondialysis patients with various degrees of kidney function (mean eGFR 50 mL/min), plasma levels of oxidized albumin were augmented progressively along with CKD deterioration [12]. In a multivariate regression model, the plasma concentration of oxidized albumin was independently and strongly inversely correlated with eGFR. In another, cross-sectional study OS biomarkers such as plasma levels of 8-isoprostanes and serum total antioxidant capacity (TAC) increased gradually along with deterioration of renal function in patients with CKD stages 1–4 and eGFR was a strong and independent predictor of 8-isoprostanes levels, after adjustment for several confounding factors [13]. However, these results are not in line with the findings by Oberg et al. who reported no correlation between F2-isoprostane levels and eGFR in a cohort of 60 patients with moderate-to-advanced CKD (stages 3–5). The relatively small number of subjects and the distribution in each stage (60% were in stages 4-5) could be identified as a serious limitation of the latter study [14]. Another prospective cohort study assessed changes of several markers of OS and inflammation prior and post renal transplantation in 19 patients. Plasma levels of C-reactive protein (CRP), tumor necrosis factor-*α* (TNF-*α*), interleukin-6 (IL-6), F2-isoprostanes, and protein carbonyls were significantly elevated compared to healthy controls before transplantation and were dramatically decreased after renal transplantation. Therefore, the authors suggested that restoration of kidney function by renal transplantation could play a beneficial role in the suppression of chronic inflammation and OS due to ESRD and maintenance renal replacement therapy [15].

OS accompanies CKD even in the early stages, progresses along with deterioration of kidney function, and becomes more severe in ESRD. OS, CKD, and inflammation are tightly interrelated and may lead to further CKD progression.

## 3. Hemodialysis and OS

Altered dietary restrictions and preferences may exaggerate the depletion of antioxidant defense mechanisms, such as low levels of vitamins C and E (mainly because of dietary restrictions of vegetables and fruits, malnutrition, and loss of vitamins during HD procedure), reduced selenium levels, and reduced function of the GSH-scavenging mechanism [16, 17]. Moreover, several factors accelerating prooxidant activity in this group of patients have been demonstrated, including the chronic excessive inflammatory status of CKD, the uremic environment, other commonly encountered characteristics of HD patients (such as hypertension, diabetes, obesity, dyslipidemia, advanced age, and enhanced vascular calcification), and factors related to the HD procedure per se [1].

There is a growing body of evidence suggesting that HD is characterized by excessive OS status, which results from loss of antioxidants during dialysis procedures and accumulation of oxidative products (Table 1). Chen et al. suggested that the HD procedure promotes the formation of O_2_^−^, a powerful prooxidant reactive oxygen molecule [10], and another study showed a direct increase in ROS plasma levels after each HD session [18]. During HD, blood exposure to dialyzer membranes and dialysate trigger activation of complement factors, platelets and polymorphonuclear white blood cells (PMNs), and subsequently ROS production, within minutes after initiation of HD sessions [16, 18–24]. PMN stimulation was reported as a significant OS biomarker that is progressively increased along with the stages of CKD and is more pronounced in HD [25]. Maher et al. reported that within 30 minutes of HD initiation, lipid peroxidation products increase and hypothesized that complement factor activation or production of free fatty acids induced by heparin might be the pathophysiologic mechanisms underlying these effects [19]. Loughrey et al. recruited 15 patients on regular HD and 15 ESRD patients with eGFR < 15 ml/min that were managed with supportive care without renal replacement therapy. Compared to the ESRD group, HD patients presented significantly higher levels of lipid peroxidation markers (MDA) and reduced plasma concentrations of antioxidants (vitamin C, GSH-Px, and selenium), indicating that OS is further exacerbated by the HD procedure [24]. Furthermore, Nguyen-Khoa showed that the duration of HD treatments is a significant independent factor of OS [26]. This observation may be counterintuitive, given that prolonged dialysis session, such as extended overnight, reveals beneficial outcomes.

HD patients are characterized by increased inflammation and lipid peroxidation [27]. The HD process promotes the formation and accumulation of oxidative products through activation of platelets, complement, and PMNs. After HD, serum ROS were 14 times higher than before the start of the HD session in a cohort of 80 chronic stable maintenance HD patients [28]. Two studies showed that ROS plasma levels were significantly increased in HD patients compared to healthy controls [29, 30]. To explain this finding, Granata et al. suggested that dysfunction of the mitochondrial respiratory system, which is prominent in CKD patients and further impaired in HD patients, might be the cause of ROS generation [30]. Handelman et al. showed that plasma levels of F2-isoprostanes were significantly higher in HD patients than in controls with normal renal function. Moreover, F2-isoprostanes were strongly associated with CRP values in the HD group, suggesting thus a tight link between OS and inflammation in patients undergoing maintenance HD [31].

## 4. Modifiable Factors Aggravating OS in HD Patients

### 4.1. Not Dialysis-Related (Lifestyle) Factors

In CKD and HD patients, lifestyle and dietary habits play a crucial role on OS status, independently from renal failure and HD-related oxidative burst.

#### 4.1.1. Diet

In a study by Bergesio et al., patients with advanced CKD on vegan diet not only had better lipid profile parameters but also had decreased OS and inflammation status compared to patients with the same level of renal function on conventional diet. The authors concluded that vegan diet might have a beneficial protective role against CV events in this particular group of patients [32]. Several studies showed that Mediterranean diet has a significant protective effect on lipid profile, inflammation, and lipid peroxidation in patients with advanced CKD, while dietary glycemic load triggered the activation of inflammatory and OS mediators in HD patients [33, 34]. A recent meta-analysis of several cohort studies showed that in CKD patients—especially ESRD undergoing HD—healthy dietary interventions, including Mediterranean, vegan, low carbohydrate diets, may have a beneficial effect on quality of life, blood pressure, and lipid profile but their impact on OS, mortality, and CV events is yet uncertain [35, 36]. Moreover, oral supplementation of fermentable dietary fiber, flaxseed, or virgin argan oil improved oxidative and systemic inflammation status in HD patients [37–40]. Therefore, healthy dietary interventions that include a balanced Mediterranean, low-carbohydrate diet with supplementation of fibers and virgin oil might be suitable for HD patients, since it showed promising and protective results against OS and inflammation. In this group of patients, larger cohort studies are required to investigate the direct effects of healthy eating patterns on OS and clinical outcomes in HD patients.

#### 4.1.2. Smoking

There are numerous studies supporting that cigarette smoking causes an increase of blood neutrophils and generates OS and inflammation in the general population [41]. Moreover, it was repeatedly shown that tobacco smoking has an additive and negative effect on serum lipid peroxidation products, as well as oxidative injury mediated by ROS in HD patients and patients with overt nephropathy [42, 43]. Due to the decreased serum levels of antioxidants such as vitamin C or total glutathione that are common in smokers [44, 45], smoking HD patients are more prone to oxidative tissue injury than nonsmoking HD subjects or even healthy individuals [26, 42, 46]. Smoking was significantly and negatively correlated with serum levels of the antioxidant total GSH in a cohort of chronic HD patients [45]. In conclusion, OS is tremendously enhanced by tobacco smoking, independently of dialysis modality, providing another reason for which smoking cessation is strongly recommended in HD patients.

#### 4.1.3. Uric Acid

During the past decades, uric acid is becoming a novel and interesting player involved in CKD and OS. Data from epidemiological studies suggest a strong association between hyperuricemia and hypertension, CV events, progression of CKD, and mortality, where OS plays a key role [47]. Furthermore, allopurinol—an inhibitor of xanthine oxidase that lowers plasma uric acid levels—was shown to act as an antioxidant in general population and CKD patients [48–50]. Regarding the possible association between hyperuricemia, OS, and poor outcomes in HD patients, the evidence is still debatable. Reducing serum uric acid levels after allopurinol administration in HD patients with metabolic syndrome resulted in significant improvement of lipid parameters and therefore might protect from future CV risk [51]. A recent study on a large cohort of 27,229 HD subjects showed that low and not high serum uric acid levels predicted all-cause and CV mortality [52]. In conclusion, the data regarding the association of hyperuricemia with OS and hard CV endpoints in HD patients is contraindicatory and scarce. Therefore, no strong recommendations regarding the lowering of uric acid in this group of patients can be made.

#### 4.1.4. Sodium and Fluid Overload

Increased sodium intake has been tightly linked with excessive OS and endothelial damage in both animal models and human studies acting through the pathway of ROS, SOD, NADPH, and NO [53–55]. In HD patients, high sodium intake and subsequent excessive fluid retention between HD sessions were significant independent predictors of all-cause and CV mortality [56], while low sodium diet was associated with prolonged survival [57, 58] and lower degree of left ventricular hypertrophy [59]. Furthermore, reduction of dialysate sodium from 140 to 137 mEq/L was accompanied by significant improvement of endothelial damage, hemodynamics, and OS status [60, 61]. In conclusion, high dietary salt intake and subsequent fluid retention in HD patients are associated with OS, endothelial injury, and poor outcomes. Therefore, strict volume control and low-salt diet are mandatory in this group of patients.

### 4.2. Dialysis-Related Factors

It has been reported that OS in HD patients is complicated by several factors, the most studied are type of dialyzer, type and dosage of heparin, medications administered, HD solution, presence of central venous catheter, and duration of HD treatment.

#### 4.2.1. Type of Dialyzer Membranes

Several investigators have reported that the type of dialysis membrane used in HD patients may play a significant role in OS production. Dasgupta et al. found that the use of the polysulphone dialyzer was accompanied by lower levels of lipid peroxidation products, compared to cuprophane [62]. Another study reported that compared with cuprophane, regenerated cellulose membrane was associated with lower production of OS markers [63]. These effects of the two types of membranes on OS production have been attributed to increased H_2_0_2_ production and water generation, caused by regenerated cellulose membranes, through catalase and GPx activities. In agreement with these findings, other investigators have shown that patients undergoing HD with cuprophane membranes exhibit significantly higher levels of ROS in monocytes and PMNs, when compared to those who dialyze with synthetic polysulphone membranes [20, 64]. Besides ROS production, several investigators studied the impact of different HD membranes on lipid peroxidation. Sevillano et al. found that HD with cuprophane membrane caused an increase in red blood cell MDA concentrations whereas the levels decreased during a HD session with the cellulose-acetate membrane [65]. Kosch et al. conducted a randomized, single-blind, crossover study to determine the impact of different HD membranes on OS and endothelial dysfunction. Twelve stable MHD patients were randomized to either cuprophane or polysulphone membrane dialysis. Flow-mediated dilatation of brachial artery and plasma levels of alpha-tocopherol (AT) and ox-LDL were assessed pre- and postdialysis. In contrast to polysulphone, HD with cuprophane was accompanied by a significant reduction in both brachial artery flow-mediated dilatation and AT serum levels, suggesting that the type of HD membrane is a significant determinant of endothelial dysfunction and OS. However, ox-LDL levels remained unaffected by either treatment [66]. On the contrary, some investigators found no difference in the effects of dialysis with cuprophane versus dialysis with polysulphone membranes on ROS production [67], while others reported that polysulphone induced an OS lower than cuprophane [63]. Another study compared cellulose membranes coated with the antioxidant vitamin E and polysulphone membranes. The cellulose-diacetate membranes resulted in increased OS-induced DNA damage in leukocytes compared to polysulphone membranes, whereas DNA damage was approximately the same for vitamin E and polysulphone dialyzers [68]. Similarly, polysulphone membranes resulted in higher plasma levels of MDA and reduced GSH-Px activity and selenium plasma levels, compared with modified cellulose (hemophan) membrane in maintenance HD patients [69]. Another study showed that regenerated cellulose HD dialyzers caused a significant increase of serum MPO, AOPP, and 8-hydroxy-2′-deoxyguanosine (8-OHdG) levels, compared with polysulphone membranes in a cohort of maintenance HD patients. The authors reached the conclusion that the biocompatibility of the HD membrane has critical effects on the development of HD-derived OS [70]. Dialysis with cuprophane dialyzer resulted in greater increase of MDA and a more severe decrease of antioxidants (vitamin E, catalase) compared to HD with polysulphone membrane [71]. However, studies examining end-points such as overall survival and major cardiovascular events have not been performed in dependency of the abovementioned different dialysis membranes.

Thus, the type of dialyzer membrane used in HD is a significant determinant of OS status and may play a role in the development of endothelial dysfunction. Although several investigators studied the effects of different dialyzer membrane types on OS production, the results remain contradictory.

#### 4.2.2. Anticoagulation

Artificial surfaces contacted by blood components in HD procedures trigger activation of platelets and PMN, which release several bioactive molecules, including MPO. MPO favors the generation of ROS, leading thus to irreversible oxidation of DNA and proteins and modification of carbohydrates and lipids. During this chain reaction, there is a significant increase in accumulation, adhesion, and degranulation of PMN cells. Accumulation and adhesion of PMNs are the early key steps for complement activation, and PMN degranulation seems to be a continuous process, independent of the complement activation pathway. However, the degranulation process is highly linked with the presence of divalent calcium cations. While the effect of the type of dialyzer on OS status in HD patients has been thoroughly studied, there is limited data on the impact of anticoagulation on the development of OS and specifically the activation of PMNs. Bos et al. were the first to investigate the effect of citrate administration as alternative anticoagulation and substitute of heparin in HD on OS granule products (MPO and lactoferrin) and PMN degranulation. Heparin and citrate administration were compared in 10 stable patients undergoing maintenance HD with cellulose-triacetate membranes. Citrate abrogated MPO release to a significant degree, whereas lactoferrin release was abolished in a lesser degree. Citrate's effect was mainly explained due to its impact on calcium divalent cations. The authors also reported that degranulation started at the beginning of HD procedures and was not associated with complement activation or neutropenia [72]. Sela et al. explored the effect of heparin on OS induced during HD in 22 stable maintenance HD patients. All patients received HD treatment with and without heparin and both pre- and postdialysis plasma levels of oxidized glutathione (GSSG) and superoxide release rate from activated neutrophils were measured. Heparin administration resulted in less superoxide release and reduced plasma levels of GSSG. These results indicate that heparin may suppress the OS derived by the HD process [73]. Another randomized crossover trial compared the effect of heparin, citrate, and dalteparin on OS and PMN degranulation in 8 maintenance HD patients and showed that degranulation of PMN and platelets are taking place early on and are highly dependent on divalent calcium cations. HD with heparin and dalteparin was accompanied with severe degranulation immediately after the start of HD, and citrate completely abolished the release of MPO and platelet factor 4, that are well-known granule molecules. Furthermore, after 7 days of citrate administration, plasma levels of ox-LDL were significantly decreased compared to HD with heparin and dalteparin [74].

#### 4.2.3. Ultrapure Dialysate

Besides dialyzer bioincompatibility, it is widely accepted that white blood cell and platelet activation leading to oxidative response are significantly enhanced by microbial contaminants of HD fluid. Even slight amounts of dialysate contamination undermine the biocompatibility of HD treatment and may aggravate HD-derived amyloidosis, inflammation, atherosclerosis, and subsequent accumulation of oxidative products [75, 76]. There is a growing body of evidence that the use of ultrapure dialysate in HD patients reduces serum levels of inflammatory mediators, *β*2-microglobulin, carbonyl, and OS biomarkers and improves anemia status [77–83]. Multiple hit theory, including blood exposure to dialyzer membranes and dialysate, endotoxin amplification, administration of intravenous anticoagulation, and finally loss of antioxidants, provides currently the best explanation concerning the oxidative burst during HD sessions, explaining thus, at least partially, the discrepancies between different studies.

#### 4.2.4. Anemia—Erythropoietin and i.v. Iron Administration

In ESRD patients under HD, anemia and OS are interrelated factors associated with poor outcomes. However, their relationship is still not clear. Renal anemia has been shown to trigger accumulation of oxidative products in HD patients, while correction of anemia may improve OS status [46]. Several observational studies highlighted that increased lipid peroxidation status might be associated with renal anemia severity in chronic HD patients [84–86]. Improvement of anemia by erythropoietin (ESA) has been accompanied by significant inhibition of the oxidation process, suggesting that anemia itself might aggravate OS [84, 87]. Both ESA and intravenous iron administration are involved in the oxidative response. Several studies supported the antioxidant effects of ESA treatment [84, 87–89], while others found no positive impact of ESA on OS status [90].

Iron metabolism is a major contributor to excessive OS in HD patients. It has been repeatedly reported that intravenous (i.v.) administration of iron sucrose causes excessive production of OS [91, 92] both *in vitro* (cell cultures) and *in vivo* [31]. Intravenous iron infusion in patients with advanced CKD (stages 3 and 4) has been shown to cause OS very rapidly and independently of transferrin saturation. Within 15 to 30 minutes after iron administration, plasma and urine levels of MDA were significantly increased. Administration of NAC (N-acetylcysteine) reduced significantly OS production, but did not show any protective effect on proteinuria and renal tubular damage [91]. Similarly, intravenous iron sucrose in maintenance HD patients was accompanied by a significant increase in free iron, total peroxide, and biological markers of lipid peroxidation, which accumulated almost immediately after the start of iron administration and reached maximum levels in 30 minutes [93]. In agreement with these studies, Muller et al. found that patients receiving i.v. iron during HD session presented significantly increased production of plasma MDA and specific OS-induced DNA damage, 30 minutes after the start of iron infusion [68]. Tovbin et al. sought to determine the effect of i.v. iron on protein oxidation and inflammation in HD patients. Intravenous administration of 100 mg of iron after 3.5 hours of high-flux HD caused a 37% elevation in protein oxidation, as assessed by AOPP levels [94]. Furthermore, in maintenance HD patients treated with intravenous iron, formation and accumulation of ox-LDL have been shown to be closely associated with iron load and enhanced erythrocyte lipid peroxidation [95]. In contrast, another study found no association between plasma levels of several OS biomarkers (GPx, SOD, TAC, vitamin C, and MDA) and ferritin levels, transferrin saturation values, and intravenous iron administration in 34 chronic HD patients receiving erythropoiesis-stimulating agent (ESA) treatment [96]. These results ignited the development of less “toxic” and “aggressive” iron preparations, which are applied as glycosylated complexes through i.v. lines, promoting biocompatibility and bioavailability studies [97]. The way of intravenous administration of iron compounds in a HD session affects directly the oxidative state. Rapid or bolus intravenous administration aggravates OS in HD patients mainly due to oversaturation of transferrin and prolonged duration of iron overload [98, 99]. On the contrary, slow i.v. iron infusion may eliminate these effects, since the native antioxidant system has more time to respond to the gradual accumulation of oxidative products [100]. In agreement with this theory, Malindretos et al. reported that slow i.v. intradialytic administration of iron sucrose or iron dextran did not trigger an oxidative or inflammatory response [101]. Moreover, administration of the antioxidant NAC significantly reduced the OS that was caused by intravenous iron therapy during a HD session [102], and therefore, it might be used as an antioxidant “shield” whenever i.v. iron infusion is necessary. Although it is widely accepted that i.v. iron is strongly linked with oxidative response during HD sessions, the lack of evidence to support a direct relationship between i.v. iron and poor clinical outcomes and the undisputed beneficial role of renal anemia correction on OS, inflammation, and survival leads to a strong recommendation for cautious and properly prescribed iron treatment for anemia management in HD patients.

#### 4.2.5. HD Modality—Hemodiafiltration

Compared with standard hemodialysis, treatment with hemodiafiltration in patients undergoing maintenance HD has a significant positive effect on OS markers and inflammation status and therefore may play a protective role against atherosclerosis and CVD [103]. The beneficial protective effects of HDF against OS are due to several factors: the ultrapure dialysate, the biocompatible membranes, the hemodynamic stability and the better anemia control of the patients, and the improved clearance of middle and large molecular weight uremic molecules such as inflammatory cytokines, homocysteine and polyamines, and *β*2-microglobulin [103–106]. After a 6-month period of standard HD, Filiopoulos et al. treated 9 stable patients with postdilution hemodiafiltration for a period of 9 months and showed that at the end of the study, there was a significant increase of TAC and a significant reduction of OS markers, such as SOD and ROS. Furthermore, hemodiafiltration suppressed inflammation, as assessed by high sensitive CRP (hs-CRP) and IL-6 [107].

### 4.3. Dialysis Access

It has been suggested that extended use of central venous catheters for vascular access might be a significant prooxidative factor that favors development of inflammation and atherogenesis [108], while Weiss et al. found an increased expression of OS biomarkers and hyperplasia factors in failed arteriovenous fistulae and grafts [109]. It may be concluded that besides the HD process itself, several conditions that are common in ESRD are involved in the pathogenesis of OS in end-stage renal disease patients.

## 5. Residual Renal Function and OS

Impaired residual renal function (RRF) in ESRD patients was associated with high inflammatory activity. Moreover, eGFR was a significant independent determinant of increased inflammatory state in ESRD patients close to the initiation of renal replacement [110]. Preserved residual kidney function was linked with decreased plasma levels of lipid peroxidation products and other markers of carbonyl and OS in ESRD patients undergoing PD [111]. The authors hypothesized that this relation might be a possible explanation for the prolonged survival of PD patients with preserved RRF [112]. Although the possible association of several HD-related factors with OS have been thoroughly studied, the role of RRF regarding OS status has not yet been elucidated. Future studies are needed in order to clarify this topic.

## 6. Diabetes and OS

OS has been suggested to play a key role in the progression of micro-/macrovascular complications of diabetes mellitus (DM). DM and CKD share common underlying pathogenetic processes which lead to accumulation of free radical products. The high glucose environment in DM triggers protein glycation and oxidation [113]. The glycated proteins are further modified and oxidized and release free radical products, the advanced glycation end products (AGEs) [114]. AGE plasma levels are significantly elevated in ESRD patients and favor OS in these patients [108]. Moreover, OS-induced accumulation of AGEs is increased in HD patients, independent from blood glucose concentrations [115].

Giugliano et al. suggested that the production and accumulation of ROS in DM is the pathogenic pathway linking impaired glucose metabolism with tissue damage and therefore may lead to DM-derived vascular complications [116]. Specifically, when endothelial cells are exposed to excessive glucose levels, they are modified and produce O_2_^−^, which inactivates NO, a well-known endothelium-relaxing factor that regulates homeostasis of the vasculature, leading thus to early, subclinical atherosclerosis. Moreover, increased plasma levels of triglycerides have been reported as common features in DM and CKD leading to accelerated free radical production [117]. Ceriello et al. [118] suggested that the impaired glycemic environment caused by high glycosylated hemoglobin increases superoxide anion generation and subsequently alters NO activity in diabetes. Dursun et al. [119] sought to investigate the effects of HD and diabetes on OS and found that both, diabetes and ESRD, induce oxidative activity. Furthermore, both conditions combined yielded synergic deleterious effects with the highest OS status, as observed in diabetics under maintenance HD. Ceriello et al. hypothesized that OS is the common pathogenic pathway linking insulin resistance with structural and functional modifications in endothelial and beta cells and subsequently leading to accelerated atherosclerosis, CVD, and DM [120]. The inter-relationship between AGEs, OS, and CVD was highlighted in a study of 225 HD patients: lipid peroxidation and glycoxidation were strongly associated with accelerated coronary vascular calcification in maintenance HD patients [121].

## 7. CVD and OS

There is a growing body of evidence that OS along with inflammation are key elements in the development and progression of vascular calcification, all-cause, and CVD mortality in patients with renal failure [108, 122, 123]. The first step of vascular calcification is the development of endothelial dysfunction. Ghiadoni et al. investigated the possible relationship between OS, CKD severity, and endothelial dysfunction in 40 CKD patients stages 3–5, 20 maintenance HD patients, and 20 healthy controls and found that flow-mediated dilatation of brachial artery was significantly reduced in CKD patients compared to controls and was further decreased in the HD group. Moreover, flow-mediated dilatation was significantly and positively related to eGFR. After oral administration of 2 g vitamin C in HD patients, flow-mediated dilatation of brachial artery was significantly elevated and OS biomarkers were reduced and therefore the authors suggested that OS and endothelial dysfunction are tightly associated in advanced CKD [124]. HD itself has been shown to trigger the accumulation of numerous oxidative factors and therefore contributes to the development of endothelial dysfunction and CVD [108], while proatherogenic molecules such as vascular endothelial growth factor and the OS marker Cu/Zn SOD were strongly associated with duration of HD treatment [125]. Similarly, duration of HD (in years) tended to be positively associated with coronary artery calcification score (CAC) in a cohort of 225 maintenance HD patients. In a multivariable model, lipid peroxides were strong predictors of CAC, independently of several traditional risk factors for atherosclerosis [121].

Although several investigators tried to explore the strong linkage between OS and progression of atherosclerosis, the exact pathophysiologic mechanisms are yet unclear. It has been reported that OS results in decrease in NO availability and subsequently causes endothelial dysfunction [126]. This affects directly the vascular tone. LDL cholesterol enters into the intima layer, where it undergoes an oxidization process and is converted into ox-LDL, a profoundly atherogenic molecule that favors the development and progression of vascular inflammation [123]. Oxidation of LDL cholesterol results in the release of MDA, a short-chain aldehyde that stimulates the expression of white blood cell adhesion and other inflammation molecules which accumulate into the subendothelial area. Tissue macrophages take up the oxidized LDL molecules in the arterial wall and form foamy cells, a key first step in the development of atherosclerosis [123]. Due to these qualitative changes in LDL, high levels of ox-LDL and increased titers of antioxidized LDL antibodies [127], along with increased concentrations of lipid peroxidation markers such as MDA [128] have been found in HD patients. Morena et al. showed that high density lipoprotein (HDL cholesterol) loses the protective ability to abrogate the LDL oxidation in HD patients and therefore may be a promoter of HD-mediated atherosclerosis [129] while Usberti et al. showed that the degree of CVD was tightly linked with the severity of lipid peroxidation in maintenance HD patients [130]. In a cohort of 32 maintenance HD and 39 continuous ambulatory peritoneal dialysis patients, MDA was negatively correlated with cardiac function—assessed by ejection fraction—and the antioxidant SOD was significantly negatively associated with systolic and diastolic blood pressure, suggesting thus that HD-induced OS may play a role in the development of left ventricular hypertrophy [131].

The primary endogenous inhibitor of NO synthase that is involved in endothelial dysfunction is asymmetric dimethylarginine (ADMA) which is increased in ESRD, possibly due to its renal secretion. High intracellular ADMA levels result in significant reduction of NO regeneration and have been repeatedly linked to endothelial dysfunction and atherosclerotic risk in both the general population and HD patients [132–134]. OS attenuates the function of the specific enzyme converting ADMA into citrulline resulting in high intracellular ADMA levels. As expected, ADMA was negatively associated with eGFR, ranking as the third risk-predicting factor after proteinuria and hemoglobin in a cohort of 131 patients with mild to severe CKD (eGFR 8 to 77 ml/min/1.73 m^2^), [135]. Moreover, plasma levels of ADMA have been found to be six times higher in HD patients compared to healthy controls [136] and five times higher in peritoneal dialysis patients than in healthy subjects [137]. Another study showed that plasma ADMA levels were higher only in HD and renal transplant recipients compared to healthy controls and not in patients with early and advanced stages of CKD [132]. Numerous studies showed the strong link between ADMA levels and early atherosclerosis and CVD complications in HD patients. Zoccali et al. showed that ADMA levels are strong predictors of cIMT (carotid intima-media thickness) and atherosclerosis progression, independently of several well-established CVD risk factors in ESRD patients [134]. Yilmaz et al. demonstrated that ADMA plasma levels were inversely correlated with eGFR and were independent, strong predictors of endothelial dysfunction in patients with CKD stages 1–5 [11]. Another study reported that increased plasma levels of ADMA are tightly linked with left ventricular hypertrophy in a cohort of 198 stable maintenance HD patients [138]. Plasma concentration of ADMA was also a strong independent predictor of cardiovascular events, all-cause, and cardiovascular mortality in a cohort of 225 ESRD patients [135, 139]. Moreover, in mild-to-severe CKD, ADMA plasma levels predicted progression to HD and all-cause mortality independent of several traditional risk factors including eGFR, hemoglobin, proteinuria, and serum CRP [135]. Therefore, there is a growing body of evidence suggesting that ADMA might be a novel risk factor for mortality and cardiovascular events in HD patients [135, 139, 140].

Besides lipid peroxidation, ADMA, and MDA, several other biomarkers of OS have been linked with development of atherosclerosis in dialysis patients. Chronic accumulation of advanced oxidation protein products (AOPPs) have been associated with high values of cIMT [141] and CVD [142]. More interestingly, Liu et al. reported a strong causal relationship between elevated plasma AOPP levels, inflammation, and atherosclerosis in animal models [143]. Dursun et al. reported that in HD patients, OS status assessed by high levels of oxidative markers (plasma TBARS) and low levels of antioxidants (catalase and plasma sulfhydryl activity) was a strong and significant independent predictor of cIMT [144]. OS-mediated DNA damage was also reported as a significant independent predictor of all-cause mortality in a cohort of 220 stable maintenance HD patients, potentially due to its effect on vascular calcification [145].

OS is tightly associated with the development and progression of atherosclerosis in CKD and HD patients. A biomarker of OS, ADMA is an inhibitor of NO synthase. ADMA is a strong predictor of atherosclerosis, mortality, and major adverse cardiac events in CKD and HD patients.

## 8. Inflammation and OS

Both enhanced OS and inflammation status are well-known interrelated factors in ESRD with common underlying mechanisms including endothelial dysfunction and common complications, such as CVD and death. It has been hypothesized by several investigators that OS causes inflammation, and on the other hand, chronic inflammation might also stimulate an oxidative response. The inflammatory status and HD duration were reported as determinants of OS in stable maintenance HD patients [26, 31], and F2-isoprostanes, well-known markers of OS, were strongly and independently associated with CRP in HD patients. Moreover, it has been shown that acute phase proteins were significantly associated with OS status in ESRD patients [146]. The exact pathophysiologic mechanisms underlying the link between OS, inflammation, and endothelial dysfunction in ESRD patients are yet unclear, although activation of neutrophils, myeloperoxidase secretion, and dysregulation of the NO system have been hypothesized as players linking these disorders [147, 148].

## 9. Hypoalbuminemia, *β*2-Amyloid Arthropathy, and Sleep Disorders

Several studies have shown that low serum albumin levels reflect poor nutritional status and are strong predictors of all-cause and CVD mortality in maintenance HD patients [149]. Danielski et al. investigated the possible association between hypoalbuminemia, chronic inflammation, and OS biomarkers in a cohort of patients undergoing HD. Plasma levels of IL-6, CRP, protein carbonyl formation, and protein thiol oxidation were significantly increased in severely hypoalbuminemic HD patients compared to normoalbuminemic. Therefore, the authors proposed that the combined additive effect of hypoalbuminemia, OS, and inflammation might result in increased CVD morbidity and mortality in maintenance HD patients [150]. Chronic accumulation of oxidative products leads to *β*2-microglobulin amyloid arthropathy. Enhanced OS and accumulation of AGEs in DM and ESRD is a key factor for the formation of amyloid fibrils [115]. OS has been associated with sleep disorders in HD patients. A recent study in 37 patients undergoing HD reported that severe sleep apnea syndrome is associated with accelerated OS status reflected by increased plasma levels of MPO and ox-LDL [151]. Chen et al. reported that improvement of sleep quality, anxiety, and fatigue in HD patients was accompanied by a significant reduction in inflammation and OS markers [152].

## 10. Antioxidants and OS in CKD and HD

The HD procedure per se is characterized by a significant depletion of antioxidants. Bayes et al. found reduced serum levels of vitamin E in HD patients [27]. Morena et al. found that patients undergoing chronic hemodiafiltration had significantly lower plasma levels of vitamin C, compared to healthy controls. Serum concentration of vitamin E did not differ significantly among these groups. Furthermore, Morena et al. quantified the exact loss of vitamin C during a hemodiafiltration session and showed that vitamin C deficiency was associated with increased levels of several oxidants (MDA, AOPP) and reduced activity of the antioxidant GSH-Px [44]. Therefore, it has been speculated that the administration of antioxidants such as vitamins E and C might be of benefit in HD patients*. In vitro*, vitamin E is the most powerful lipid-soluble antioxidant molecule in cell membranes. It not only preserves the stability of biological membranes and protects them from injury induced by ROS and lipid peroxides but it also modifies the cell reaction to OS via regulation of signal-transmission molecular pathways [153]. Moreover, in salt-sensitive hypertensive rats, combined therapy of vitamins E and C ameliorated the accumulation of oxidative products, improved kidney hemodynamics, and subsequently protected the kidney from further damage [154]. However, the clinical data regarding the use of oral vitamin supplements for antioxidant protection in HD remains controversial and does not preclude vitamin E supplementation for HD patients (Table 2).

### 10.1. Vitamin C Supplementation and OS

Several investigators have reported no effect of oral or intravenous administration of vitamin C on various markers of OS (plasma levels of TBARS and isoprostanes, Cu/Zn activity, and LDL susceptibility to oxidation), in patients undergoing maintenance HD [155–158]. Fumeron et al. conducted a prospective, randomized open-label trial to investigate the possible effects of oral vitamin C administration on inflammation and OS biomarkers in a cohort of maintenance HD patients. Oral vitamin C (250 mg, thrice weekly) was given in 33 stable HD patients for two months. Although serum levels of vitamin C and ascorbate were normalized by oral supplementation, serum levels of CRP, albumin, carbonyls, or concentrations of reduced and oxidized glutathione in RBCs remained unchanged [159]. The investigators proposed the short period of treatment (2 months) and the route of administration (oral instead of intravenous) as possible explanations for their findings. In disagreement with the previous studies, Ghiadoni et al. reported that oral administration of 2 g vitamin C reduced OS biomarkers such as plasma MDA, lipoperoxides, and increased plasma antioxidant capacity (assessed by ferric reducing ability of plasma) in both HD and CKD stage 3 and 4 patients [124]. Two other randomized, placebo-controlled studies showed that 250 mg/day oral intake of vitamin C in HD patients reduced plasma and RBC MDA levels, although one study showed marginally nonsignificant effects [160, 161], while another study reported that intravenous administration of vitamin C during HD sessions significantly decreased HD-mediated OS in a study of 80 HD patients [28].

### 10.2. Vitamin E Supplementation and OS

Three randomized controlled trials showed no positive effect of vitamin E intake on OS development. Firstly, Diepeveen et al. administered 800 IU/day alpha-tocopherol for 12 weeks in a cohort of 23 HD and 21 PD patients and found that vitamin E did not alter plasma ox-LDL levels [162]. Secondly, Lu et al. showed that oral therapy with 800 IU of vitamin E every day for 6 months had no effect on plasma oxidative protein levels in stable HD patients compared to HD patients that received placebo [163]. Similarly, Kamgar et al. reported that after 8 weeks of daily oral treatment with a combination of vitamins (250 mg vitamin C, 800 IU vitamin E, 100 mg vitamin B_6_, 250 *μ*g vitamin B_12_, and 10 mg folic acid), the plasma levels of protein carbonyls, F2-isoprostanes, IL-6, and CRP remained unchanged and suggested that oral antioxidant multivitamin therapy does not improve OS, inflammatory, nutritional, and erythropoiesis status in maintenance HD patients [164]. In agreement, three open-label studies performed with small numbers of HD patients with and without DM2 failed to show any beneficial effect of oral vitamin E intake on plasma levels of isoprostanes, autoantibodies against ox-LDL and phosphatidylcholine hydroperoxide (PCOOH), [165–167]. One study in a relatively small cohort of subjects showed that prolonged (for one-year period), daily oral supplementation of 500 mg vitamin E in 27 HD patients resulted in a decrease in serum concentrations of some antioxidants (SOD, TAC) compared to the placebo HD group [168].

Several investigators suggested that vitamin E administration might have a protective role against OS in HD patients. Vitamin E intake has been shown to decrease membrane lipid peroxidation of platelets and red and white blood cells in HD patients [169]. Two small studies reported a beneficial effect of oral vitamin E intake on erythrocyte MDA levels and lipid peroxidation status in HD patients [170, 171]. Another study found that high daily oral intake of vitamin E for 2 months caused a decrease in serum ADMA levels in CKD predialysis patients [172]. Three open-label studies in HD patients reported that daily oral intake of AT (500–800 mg/day) resulted in significant reduction of plasma TBARS and induced antioxidant plasma levels of GSH, GPx, and SOD [173–175]. Domenici et al. reported that supplementation of vitamin E in both PD and HD patients resulted in decrease of protein oxidation and reduction of OS-induced DNA damage [176]. Several studies have suggested that vitamin E intake improves erythropoiesis factors by suppressing OS development in HD patients. The beneficial effect of oral vitamin E intake on erythrocyte fragility was first reported by Ono. Oral administration of 600 mg vitamin E every day for a month resulted in significant improvement of anemia, RBC fragility, and both serum and RBC vitamin E concentrations in a cohort of stable HD patients [177]. Oral administration of vitamin E improved renal anemia and lowered requirements of ESA [178] and had a protective role against OS induced by intravenous iron administration [93] in HD patients. Another study showed that combined treatment of ESA with oral intake of vitamin E (15 mg/kg daily) resulted in a significant reduction of the OS marker GSSG/GSH ratio and a considerable improvement in erythropoiesis compared to ESA therapy alone in children receiving chronic HD [179]. Uzum et al. conducted a randomized controlled trial and showed that treatment with 300 mg vitamin E daily for a period of 20 weeks resulted in a significant decrease of erythrocyte osmotic fragility and lipid peroxidation—as assessed by plasma MDA—in HD patients [180]. Hodkova et al. [181] reported that combination of oral AT and intravenous iron administration reduced PMN respiratory burst after iron intake in a cohort of HD patients. Therefore, vitamin E might be a beneficial supplement that suppresses the immunologic preoxidative activity induced by HD and iron infusion. However, it has to be cautioned, given that in other randomized studies, vitamin E supplementation yielded a negative result, that is, the supplementation led to no difference in cardiovascular events when supplemented over 4 years [182].

Although there is accumulating data suggesting that supplementation of various antioxidants such as vitamins C + E and NAC might reduce OS state in HD, the studies available are not consistent. The inconsistency between interventional studies aiming OS reduction by antioxidant supplementation in HD patients is due to several factors: firstly, OS status is assessed by numerous different biomarkers in different timelines and in heterogonous cohorts of patients; secondly, the dosage and the supplementation pathway of antioxidants differs between the trials; thirdly, the number of patients studied is relatively small to support strong conclusions; and finally, the exact pathophysiologic mechanism and the degree of OS abrogation by antioxidants is yet unclear. Although common antioxidants such as vitamins C + E seem to exert significant antioxidant effect on HD patients in small dosages, when they are administered in high dosages not only they lose their protective effect but it has been reported that they might actually act as prooxidants [168]. For all these reasons, there is a discrepancy between interventional studies regarding antioxidant supplementation and OS inhibition as well as patient adverse outcomes in HD patients. Therefore, antioxidant intake has not yet been adopted in guidelines or everyday clinical practice.

### 10.3. N-Acetylcysteine (NAC) and OS

NAC is a well-known thiol-containing free radical scavenger that induces cysteine and glutathione production. NAC exerts significant anti-inflammatory actions and is widely used as a pharmacologic antioxidant. NAC has the ability to scavenge ROS directly leading to production of cysteine, which triggers the release of glutathione, a powerful antioxidant [183]. NAC has been used for the treatment of several conditions related to OS, such as bronchiolitis and paracetamol overdose, whereas it has been shown to exert protective effects in the preservation of renal function in acute kidney injury and CKD [184–187]. Moreover, Trimarchi et al. reported that oral administration of NAC results in a significant reduction of MDA levels possibly through elevation of glutathione concentrations [188] and Witko-Sarsat et al. showed that NAC successfully decreased AOPP-derived responses of both normal and uremic neutrophils [189]. Another randomized placebo-controlled, double-blinded study showed that intravenous administration of high dose NAC (5 g) resulted in significant reduction of serum ADMA levels, compared to the placebo group [190]. Swarnalatha et al. [102] conducted a randomized placebo-controlled study and divided 28 HD patients that were treated with iron infusion in two groups: 14 were given NAC and 14 placebo. The NAC group showed reduced plasma levels of MDA compared to the placebo group. In a very similar study, Garcia-Fernandez et al. treated 80 HD patients with intravenous administration of 2 g of NAC and divided them in two groups: 40 received 50 mg of iron sucrose and the remainder 40 patients received 100 mg during HD. NAC resulted in significant increase in TAC in both groups, whereas MDA serum levels were only reduced in the low iron dose group [191].

Several studies showed that oral or i.v. administration of NAC—a powerful antioxidant scavenger—result in significant reduction of OS status in HD patients.

### 10.4. Statins and OS

Several investigators sought to determine the possible protective effects of statins and eicosapentaenoic acid (EPA)—a polyunsaturated omega-3 fatty acid—on the formation and accumulation of oxidized, atherogenic lipoproteins in patients undergoing renal replacement therapy. Ando et al. randomized 22 HD patients to either 1.8 g of EPA or placebo for 3 months and found that EPA supplementation significantly decreased plasma levels of ox-LDL and atherogenic remnant lipoproteins [192]. Nishikawa et al. showed that daily treatment with 5 mg of simvastatin for 6 months improved several lipid profile parameters and reduced the levels of MDA in 38 HD patients [193]. In contrast, Martinez-Castelao and coworkers [194] showed no effect of fluvastatin treatment on LDL susceptibility to oxidization in renal transplanted patients with dyslipidemia. The first prospective randomized, double blind placebo-controlled trial of treatment with statin and vitamin E in HD patients showed that the use of statins improved significantly the lipid profile and ox-LDL levels and might prevent CVD complications in these patients. Additional administration of vitamin E did not influence any lipid parameters but significantly reduced *in vitro* LDL oxizability, likely acting synergistically with the statin treatment [162]. Data from the LORD study suggest that administration of atorvastatin 10 mg/day for 3 years had no effect on plasma levels of F2-isoprostanes, protein carbonyls, glutathione peroxidase activity, and total antioxidant capacity in CKD patients [195].

Taken together, the possible antioxidant effects of statins in HD patients are not unequivocally shown.

### 10.5. Antioxidants and CVD

Although many investigators published promising results about the potential beneficial effects of oral/intravenous antioxidants on OS and inflammation status, the data regarding the impact of antioxidants on mortality and CVD events is scarce. Ghiadoni et al. reported that oral administration of 2 g of vitamin C seemed to improve significantly the endothelial dysfunction status—assessed by brachial artery endothelium-dependent vasodilation to reactive hyperemia and the response to sublingual glyceryl trinitrate—in HD but not in CKD patients [124]. Ono found no effect of vitamin C administration on morbidity and mortality in a cohort of 61 HD patients, after a period of 2 years [196]. In disagreement, Boaz et al. conducted a randomized double-blinded, placebo-controlled study in HD patients with previous history of CVD. The patients were randomized into two groups: 97 received 800 IU per day of natural vitamin E and 99 received placebo. After a follow-up period of 519 days, the authors reported that the vitamin E group presented a significant 70% reduction in myocardial infarction and 40% in composite CVD end-points [197]. Another large randomized double-blinded, placebo-controlled study showed significant decrease in composite cardiovascular end-points in HD patients that were treated with NAC compared to the control group of HD subjects that received placebo [198]. Himmelfarb et al. conducted a large prospective, double-blinded, placebo-controlled randomized trial (provision of antioxidant therapy in hemodialysis (PATH) trial) to investigate the effects of oral antioxidant treatment administered for 6 months on biomarkers of OS, inflammation, and erythropoiesis. The study included 353 patients undergoing maintenance HD that were randomized to treatment with a cocktail of tocopherols (666 IU per day) and *α*-lipoic acid (600 mg per day) or placebo. Plasma levels of hs-CRP, IL-6, F2-isoprostanes, and isofurans were assessed at baseline (similar concentration for all biomarkers measured) and at 3 and 6 months. The authors failed to show any significant differences for circulating levels of OS biomarkers between the two groups. Furthermore, during the 6-month period, the hospitalization and all-cause mortality rates were similar between the two groups [199]. A recent systematic review and meta-analysis included 10 randomized controlled studies that explored the possible effects of antioxidant agents (vitamins A, C, and E and NAC) on mortality, CVD, and CKD progression in 1979 patients with CKD stages 3–5, renal transplant recipients, and on HD (June 2012, 2013). Although the use of antioxidants failed to prevent all-cause/CVD mortality and CVD morbidity (coronary heart disease, cerebrovascular disease, and peripheral artery disease) in CKD, antioxidant treatment in HD patients exhibited significant protection against CVD events. Moreover, in the nondialysis group (404 patients with CKD 3 + 4 and renal transplant recipients), administration of antioxidants delayed the progression of ESRD and was associated with preservation and even increase of eGFR [200, 201].

Thus, antioxidant administration may play a significant protective role against death and major adverse cardiac events in HD patients and prevents progression of ESRD in CKD patients.

## 11. Vitamin E-Coated Membranes and OS

Vitamin E has been repeatedly shown to be a scavenger of lipid hydroperoxides involved in the regulation of lipid oxidation *in vitro* [202] and a significant antioxidant and antiatherogenic molecule *in vivo*. Based on the fact that the interaction of blood with the dialyzer triggers the production of prooxidants, the use of vitamin E as adjunct in the membranes to additively provide a scavenger seemed an interesting approach. Galli et al. were the first to report that coating the blood surface of HD filters with the antioxidant vitamin E retained blood antioxidants (especially vitamin E) and prohibited the lipoperoxidation process both *in vitro* and *in vivo* and therefore could be considered a profound biocompatible material [203]. Moreover, the same group found that vitamin E-coated membranes (VECM) prevented efficiently the production of free radicals (and particularly ROS) by phagocytic neutrophils *in vitro* and *in vivo* [23]. They suggested that administration of vitamin E abrogates lipid peroxidation, protects serum GSH and thiols from the oxidation process, and seems to play a pivotal role in the modification of the NO metabolism [174, 204]. The beneficial effects of VECM besides their antioxidant activity lie on their ability to inhibit complement activation, a common side-effect of the less biocompatible generated cellulose membranes. VECM cellulose showed significantly reduced activation of both mononuclear cells and the complement pathway compared to cellulose acetate dialyzers [205]. Another crossover study showed that compared to polyamide dialyzers, VECM presented similar effects on lymphocyte function, but additionally, it suppressed the formation of proinflammatory cytokines [206]. This may be due to the fact that vitamin E abrogates directly the release of proinflammatory cytokines by white blood cells, especially monocytes [207]. Membranes coated with vitamin E showed a significant protective role in alleviating HD-mediated OS, especially when combined with intravenous administration of vitamin C in a cohort of 80 stable HD patients [28]. Another study showed that VECM resulted in a higher degree of fatty acid unsaturation in erythrocyte cell membrane [208]. Similarly, it was reported that a 3-month treatment with VECM resulted in significant improvement of serum and HDL vitamin E content that was linked with lower susceptibility of HDL to oxidation in 12 HD patients [209]. VECM was also found to suppress the peroxidation status in serum lipids and erythrocytes, to reduce the circulating levels of AGEs and to pacify the immune activity [206, 210]. Furthermore, they have been reported to play a protective role against hemodialysis-induced endothelial dysfunction and increased production of ox-LDL [184]. In another study, 10 nondiabetic and 8 diabetic patients underwent regular HD with polysulphone membrane for 1 month and then changed to treatment with VECM for 6 months. MDA, AGEs, and 8-OHdG were assessed as markers of lipid peroxidation, glycoxidation, and DNA damage, respectively. Plasma levels of MDA, AGE and 8-OHdG were significantly elevated after a polysulphone HD, while a single dialysis session with VECM completely abrogated this increase. After use of VECM for a period of 6 months, there was a significant decrease in the plasma levels of AGEs and 8-OHdG in both diabetics and nondiabetics, while plasma MDA was decreased only in diabetic patients after three months of treatment. Furthermore, the improvement of OS status was more pronounced in the diabetic group [211]. Another randomized crossover study reported that maintenance HD patients dialyzed repeatedly with vitamin E-coated membranes for 12 weeks presented significant improvement in lipid parameters, oxidative stress, and polymorphonuclear function, compared with patients treated with control dialyzer membranes, that were identical to VECM, except for AT binding [212]. The long-term beneficial effects of VECM on OS and inflammation status were evaluated by Takouli et al. who switched 9 HD patients to VECM for a period of 12 weeks and then back to the original polysulphone dialyzer for 24 weeks. At the end of the study, ROS were significantly reduced and TAC and SOD were elevated. Plasma levels of the inflammatory markers hs-CRP and IL-6 were dramatically decreased [213]. A meta-analysis of 14 studies suggested that treatment with VECM resulted in significant improvement of lipid peroxidation markers, such as TBARS and MDA [214].

Modification of the dialyzer with vitamin E might increase its biocompatibility [215]. After 2 years follow-up of 50 stable maintenance HD patients, the group that was dialyzed with VECM presented significantly lower levels of LDL malondialdehyde and ox-LDL, compared to the group that received treatment with standard cellulose membrane dialyzers. Additionally, the use of these modified dialyzers resulted in slower progression of atherosclerosis, assessed by the aortic calcification index [215]. In agreement with these results, Morimoto et al. found that long-term (6 months) dialysis treatment with VECM was accompanied by significant decrease in ADMA, ox-LDL, and MDA-LDL plasma levels compared to dialysis with polysulphone dialyzers. After the 6-month period of VECM exposure, the patients were switched to HD with polysulphone membranes for 1 year. ADMA, ox-LDL, and MDA-LDL serum levels were increased back to the baseline. Since VECM seem to exert a powerful antioxidant effect and decrease the levels of circulating ADMA (a well-known independent predictor of all-cause mortality and CVD in HD patients), it is interesting to hypothesize that VECM might prevent all-cause and CVD mortality in these patients [216]. A recent study suggested that HD treatment with VECM may play a significant protective role against OS, anemia, and vitamin E deficiency. The use of VECM was accompanied by a significant reduction in the levels of DNA damage induced by OS [217].

A very recent meta-analysis of 60 studies aimed to summarize the available data on the effects of VECM over standard HD membranes on inflammation, anemia, and OS. The data suggest that the new membranes exert positive effects on the erythropoietin resistance index. Regarding inflammation and OS status, VECM resulted in reduction of IL-6 levels, circulating MDA, and levels of TBARS, while plasma and RBC vitamin E levels were significantly increased [218]. Lipid parameters and dialysis adequacy were not altered by the use of the new membranes.

Compared to standard HD membranes, dialysis treatment with vitamin E-coated membranes may possibly confer a protective role against inflammation, OS, and erythropoietin resistance.

## 12. Conclusions

OS is a universal challenge in life and induces a counterresponse by exposed cell. The enhanced OS status that characterizes HD patients is mainly due to poor dietary intake of exogenous antioxidants, accumulation of oxidative products, and loss of antioxidant molecules during HD and is highly linked with development of atherosclerosis, chronic inflammation, and all-cause and CVD mortality in these patients. Although the administration of antioxidants seems to play a beneficial role against OS development in maintenance HD patients, it has not yet been adopted in the everyday clinical practice. Large, prospective studies are urgently needed to elucidate the possible protective role of antioxidant administration against cellular stress that hold the promise to ameliorate the cardiovascular risk profile in CKD and end-stage renal disease. It seems that OS is an undisputed component of the uremic environment and since uremia is a well-established nontraditional risk factor for CV events and all-cause/CV mortality, it is tightly linked with early atheromatosis and CV disease. Therefore, OS should be incorporated in a “uremic milieu” abnormality approach and might constitute a novel but quite important therapeutic target in chronic HD patients. Based on the available data, the best renal replacement therapy for reducing OS is HDF with ultrapure dialysate and synthetic membranes. It is not justified or safe to derive strong recommendations for either oral or intravenous antioxidant supplementation in HD patients, due to the fact that the interventional studies regarding this topic have failed to produce concrete results. However, assessing OS status in HD patients is a matter under discussion and probably in the light of more solid evidence could be incorporated in future routine clinical practice or even guidelines.

## Figures and Tables

**Table 1:** Effect of HD session on OS.

Study (ref.)	Year	OS biomarker	Patients	Results
*Data regarding effect of HD on OS*				
Sela et al. [25]	2005	MPO activity in PMNLs SOD release rate in PMNLs	30 CKD (stages 2–5) 30 CAPD 30 HD 30 healthy controls	HD > CAPD > CKD > controls
Maher et al. [19]	1987	Free radicals	51 HD 33 healthy controls	Baseline: same HD = control After 15′ of HD: ↑ After 30′ of HD: peak
Descamps-Latscha et al. [20]	1991	Complement activation and stimulation of PMNLs	20 HD	After 15′ of HD: ↑, peak
Himmelfarb et al. [21]	1993	ROS	10 HD	After 15′ of HD: ↑ (×6.5 times) After 30′ of HD: peak
Yang et al. [28]	2006	ROS Plasma vitamin C level	80 HD	After HD: ↑ (×14 times)
Clermont et al. [22]	2001	Plasma vitamin C level ascorbyl free radical/vitamin C ratio PMNL activation	16 HD	After HD: ↑↑
Chen et al. [10]	1998	SOD MPO	104 HD 98 healthy controls	Baseline: HD > control After HD: HD >>> control
Nguyen et al. [18]	1985	ROS	35 HD 44 healthy controls	Baseline: HD < control After 15′ of HD: ↑ HD >> control End of HD: ↓ HD < control

CKD: chronic kidney disease; HD: hemodialysis; OS: oxidative stress; MDA: malondialdehyde; ADMA: asymmetric dimethylarginine; SOD: superoxide dismutase; Zn: zinc; Cu: copper; Se: selenium; GSH-Px: glutathione peroxidase; TAS: total antioxidant status; ESRD: end-stage renal disease; CAPD: continuous ambulatory peritoneal dialysis; MPO: myeloperoxidase; PMNL: polymorphonuclear neutrophil; ROS: reactive oxygen species.

**Table 2:** Effect of antioxidant supplementation on OS in HD patients.

Study (ref.)	Patients	OS biomarker	Antioxidant	Study period	Result
*Vitamin C*					
Yang et al. [28]	40 on Vit. C 40 controls	ROS	i.v. 1 g	4 hours	↓ OS
Ghiadoni et al. [124]	20 on Vit. C	MDA Lipoperoxides Ferric-reducing plasma ability	p.o 2 g	4 hours	↓ OS
Fumeron et al. [159]	33 on Vit. C	Serum carbonyls, RBC concentrations of reduced and oxidized glutathione	p.o 0.25 g ×3/week	8 weeks	Same OS
Candan et al. [160]	17 on Vit. C 17 on placebo	MDA RBC osmotic fragility	p.o 0.25 g	12 weeks	↓ OS
Abdollahzad et al. [161]	21 on Vit. C 21 on placebo	MDA	p.o 0.25 g	12 weeks	↓ OS
Eiselt et al. [155]	20 on Fe i.v. 5 on Fe i.v. + Vit. C	TBARS	i.v. continuous 2 mg/min	4 weeks	↑ OS
Chan et al. [156]	10 on 250 mg p.o 11 on i.v.	F2-isoprostanes	p.o/iv	12 weeks	Same OS
Ramos et al. [157]	17 on Vit. C 17 on placebo	TBARS Lipoperoxides	p.o 1 g/d	1 year	Same OS
Washio et al. [158]	16 on Vit. C	Cu/Zn-SOD	p.o 0.2 to 1 g	3 weeks	Same OS

*Vitamin E*					
Diepeveen et al. [162]	12 on Vit. E 11 on placebo	Ox-LDL	p.o 800 IU/d	12 weeks	Same OS
Lu et al. [163]	14 on Vit. E 13 on placebo	Oxidative protein modifications Lipoperoxides	p.o 800 IU/d	24 weeks	Same OS
Kamgar et al. [164]	20 on multivitamin (including Vit. E) 17 on placebo	F2-isoprostane protein carbonyl	p.o 800 IU/d	8 weeks	Same OS
O'Byrne et al. [165]	16 on Vit. E	Ox-LDL antibodies	p.o 800 IU/d	12 weeks	Same OS
Sanaka et al. [166]	11 on Vit. E 11 on placebo	PCOOH	p.o 500 mg/d	—	Same OS
Smith et al. [167]	11 on Vit. E	F2-isoprostanes	p.o 400 IU/d	8 weeks	Same OS
Antoniadi et al. [168]	27 on Vit. E 20 on placebo	TAS RBC SOD activity GSH-Px	p.o 500 mg/d	1 year	↑ OS
Inal et al. [170]	36 on EPO (100 U/kg) 36 on 50% decreased EPO dosage + Vit. E	MDA SOD activity CAT activity	p.o 300 mg/d	12 weeks	↓ OS
Badiou et al. [173]	14 on Vit. E	Cu-induced LDL oxidation TBARS	p.o 500 mg/d	24 weeks	↓ OS
Galli et al. [174]	7 on Vit. E	GSH TBARS NO	p.o 800 mg/d	3 weeks	↓ OS
Giray et al. [175]	36 on Vit. E	GSH-Px SOD + CAT TBARS	p.o 600 mg/d	14 weeks	↓ OS
Domenici et al. [176]	29 on Vit. E	8-OHdG	p.o 300 mg/d ×3/week	4 weeks	↓ OS
Ono [177]	30 on Vit. E	RBC osmotic fragility	p.o 600 mg/d	4 weeks	↓ OS
Cristol et al. [178]	7 on ESA + Vit. E 30 control	MDA RBC SOD RBS GSH RBC Vit. E	p.o 500 mg/d	24 weeks	↓ OS ↑ Hb
Nemeth et al. [179]	10 children on ESA for 2 weeks, then ESA + Vit. E for 2 weeks	GSSG/GSH	p.o 15 mg/kg/d	4 weeks	↓ OS ↑ Hb
Uzum et al. [180]	19 on Vit. E 15 controls	MDA RBC osmotic fragility	p.o 300 mg/d	20 weeks	↓ OS
Hodkova et al. [181]	7 on i.v. iron + Vit. E	AOPPs PMNLs burst	p.o 200 mg/d	7 days	Same OS

*NAC*					
Swarnalatha et al. [102]	14 on NAC 14 on placebo	MDA	p.o 600 mg ×2/day Prior to i.v. iron	10 days	↓ OS
Garcia-Fernandez et al. [191]	10 iron 50 10 iron 50 + NAC 10 iron 100 10 iron 100 + NAC	MDA	i.v. 2 g Prior to i.v. iron	10 days	↓ OS
Trimarchi et al. [188]	12 on NAC 12 control	MDA	p.o 600 mg ×2/day	30 days	↓ OS
Witko-Sarsat et al. [189]	16 HD Cells incubated with NAC (in vitro study)	Serum albumin AOPPs	2 mg/mL	30′	↓ OS
Thaha et al. [190]	20 on NAC 20 on placebo	ADMA	i.v. 5 g	4 hours	↓ OS

*Statins*					
Diepeveen et al. [162]	12 on atorvastatin 11 on placebo	Ox-LDL	p.o 40 mg/d	12 weeks	↓ OS 30–43%
Ando et al. [192]	11 on EPA 11 on placebo	Ox-LDL	p.o 1.8 g/d	12 weeks	↓ OS 38%
Nishikawa et al. [193]	38 on simvastatin	MDA	p.o 5 mg/d	24 weeks	↓ OS

RBC: red blood cell; TBARS: thiobarbituric acid-reactive substances; Ox-LDL: oxidized low-density lipoprotein; PCOOH: phosphatidylcholine hydroperoxide; EPO: erythropoietin; CAT: catalase; NO: nitric oxide; 8-OHdG: 8-hydroxy 2′-deoxyguanosine; AOPPs: advanced oxidation protein products; GSSG: oxidized glutathione; EPA: eicosapentaenoic acid.

## Abbreviations

8-OHdG: 8-Hydroxy-2′-deoxyguanosine
ADMA: Asymmetric dimethylarginine
AGEs: Advanced glycation end products
AOPPs: Advanced oxidation protein products
AT: Alpha-tocopherol
CAC: Coronary artery calcification score
CIMT: Carotid intima-media thickness
CKD: Chronic kidney disease
CVD: Cardiovascular disease
DM: Diabetes mellitus
EGFR: Estimated glomerular filtration rate
EPA: Eicosapentaenoic acid
ESA: Erythropoiesis-stimulating agent
ESRD: End-stage renal disease
GDPs: Glycose degradation products
GSH-Px: Glutathione peroxidase
GSH: Reduced glutathione
GSSG: Oxidized glutathione
H_2_O_2_: Hydrogen peroxide
HD: Hemodialysis
HDL: High-density lipoprotein
Hs-CRP: High-sensitive C-reactive protein
IL-6: Interleukin-6
i.v.: Intravenous
LDL: Low-density lipoprotein
MDA: Malondialdehyde
MPO: Myeloperoxidase
NAC: n-Acetylcysteine
NO: Nitric oxide
O_2_^−^: Superoxide anion radical
ONOO^−^: Peroxynitrite
OS: Oxidative stress
Ox-LDL: Oxidized LDL
PCOOH: Phosphatidylcholine hydroperoxide
PD: Peritoneal dialysis
PMNs: Polymorphonuclear white blood cells
RBC: Red blood cell
ROS: Reactive oxygen species
RRF: Residual renal function
SOD: Superoxide dismutase
TAC: Total antioxidant capacity
TBARS: Thiobarbituric acid-reactive substances
TNF: Tumor necrosis factor
VECM: Vitamin E-coated membrane.
